# Can Simply Answering Research Questions Change Behaviour? Systematic Review and Meta Analyses of Brief Alcohol Intervention Trials

**DOI:** 10.1371/journal.pone.0023748

**Published:** 2011-10-05

**Authors:** Jim McCambridge, Kypros Kypri

**Affiliations:** 1 Department of Public Health & Policy, Centre for Research on Drugs & Health Behaviour, London School of Hygiene & Tropical Medicine, London, United Kingdom; 2 Centre for Clinical Epidemiology and Biostatistics, School of Medicine and Public Health, University of Newcastle, Newcastle, Australia; University of Oxford, United Kingdom

## Abstract

**Background:**

Participant reports of their own behaviour are critical for the provision and evaluation of behavioural interventions. Recent developments in brief alcohol intervention trials provide an opportunity to evaluate longstanding concerns that answering questions on behaviour as part of research assessments may inadvertently influence it and produce bias. The study objective was to evaluate the size and nature of effects observed in randomized manipulations of the effects of answering questions on drinking behaviour in brief intervention trials.

**Methodology/Principal Findings:**

Multiple methods were used to identify primary studies. Between-group differences in total weekly alcohol consumption, quantity per drinking day and AUDIT scores were evaluated in random effects meta-analyses.

Ten trials were included in this review, of which two did not provide findings for quantitative study, in which three outcomes were evaluated. Between-group differences were of the magnitude of 13.7 (−0.17 to 27.6) grams of alcohol per week (approximately 1.5 U.K. units or 1 standard U.S. drink) and 1 point (0.1 to 1.9) in AUDIT score. There was no difference in quantity per drinking day.

**Conclusions/Significance:**

Answering questions on drinking in brief intervention trials appears to alter subsequent self-reported behaviour. This potentially generates bias by exposing non-intervention control groups to an integral component of the intervention. The effects of brief alcohol interventions may thus have been consistently under-estimated. These findings are relevant to evaluations of any interventions to alter behaviours which involve participant self-report.

## Introduction

The contribution of behavioural risk factors, such as physical inactivity, tobacco smoking, and unhealthy alcohol use, is estimated to be at least 20% of the total global burden of disease [1]. Accordingly there is increasing investment in the development of behavioural interventions. Attempts to influence behaviour have also gained a new prominence in wider public policy, for example in efforts to combat climate change and domestic terrorism. Trials and other evaluation studies typically involve asking study participants about their own behaviour over time, which in some cases may be validated with objective measures. Such data are fundamental to the behavioural sciences [2]. This process of reporting on one's own behaviour may itself induce reflection and actual change and this was the original reason for the introduction of control groups in behavioural research a century ago [3]. The Hawthorne effect, wherein participants change their behaviour in response to being monitored, has been widely discussed for three quarters of a century [4], [5], [6] and has entered “the folklore of behavioural science” [7]. Accounts of unexpected improvements apparently due to research assessments are often invoked as possible explanations for null findings in trials across a wide range of behaviours (see for example [8]). As the technological capacity for monitoring behaviour grows, for example through the use of pedometers in relation to walking, so does the need to better understand this phenomenon [9].

Longstanding recognition of the Hawthorne effect and the possible implications of answering questions in the context of research study assessments have not, however, led to any substantial tradition of experimental study in health sciences or elsewhere. Alongside some interesting non-experimental studies [10], [11], [12], there exist somewhat isolated trials of the effects of questionnaire completion on disparate health outcomes [13], [14], [15], [16], [17], [18], [19], [20], [21]. This situation has changed recently in the field of brief alcohol intervention trials in which individualised feedback, advice and brief counselling are evaluated for public health benefit [22], [23]. Assessment effects may have greater bias potential in these studies because of similarities with the evaluated interventions, which invariably require assessment, and because effect sizes are themselves small, their value deriving from potential for wide dissemination [24]. Assessment effects and brief interventions may also operate by similar mechanisms, acting upon the self-regulation of behaviour [21]. There have been no systematic reviews which investigate whether answering questions on a particular behaviour, which may be intrinsic to intervention study, subsequently impacts upon that behaviour. The objective of the present study is therefore to evaluate the size and nature of effects observed in randomized manipulations of the effects of answering questions on drinking behaviour in the non help-seeking populations who participate in brief intervention trials.

## Methods

### Study design & data collection

We excluded assessments undertaken with the specific purpose of changing behaviour, as these were judged likely to involve additional components, which may or may not have been reported. We are thus studying the effects of research assessments only. Peer-reviewed journal publications in any language were included and studies undertaken in alcohol treatment services excluded. There were no other selection criteria. This review has been reported in accordance with the PRISMA statement and was undertaken without a published protocol [25].

**Figure 1:**
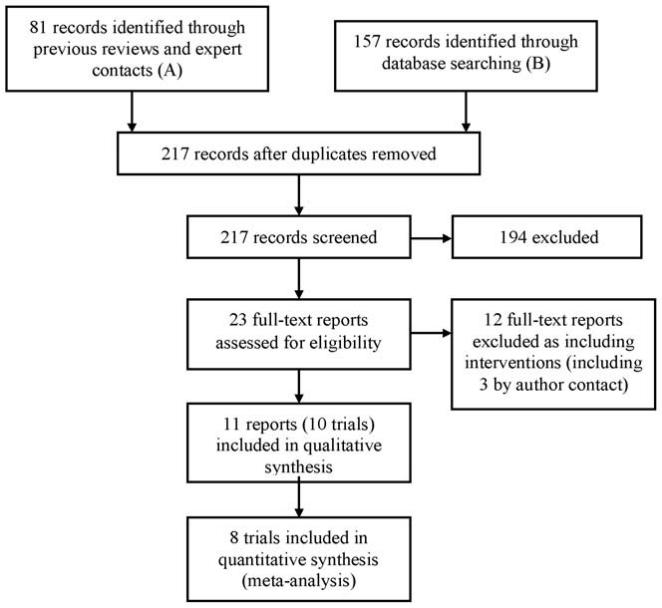
Participant flowchart.

There have been many reviews of the brief alcohol intervention literature and we used these to identify relevant studies for this review (‘A’ in Figure 1). We contacted experts both individually and via three groups, the International Network on Brief Interventions for Alcohol Problems (INEBRIA), the Kettil Bruun Society for Social and Epidemiological Research on Alcohol, and the Research Society on Alcoholism (also included in ‘A’ in Figure 1). We searched PubMed using the terms “assessment” AND “alcohol” AND “reactivity”, with the final database searches taking place on 8^th^ February 2011. The flowchart in Figure 1 summarises this process. Nine studies were excluded when the reports revealed the presence of non-assessment intervention components. Finally, three studies were excluded when author contact ascertained that assessments were undertaken specifically for intervention purposes [26], [27], [28].

### Outcomes & analyses

Various outcome measures are used in this literature. *A priori* we decided to select outcomes for quantitative study according to their availability: (1) overall total alcohol consumed within the past week or a typical recent week was reported or could be derived in all studies which provided quantitative data; (2) quantity consumed per drinking day was missing in only two cases; and (3) the WHO Alcohol Use Disorders Identification Test [29] (AUDIT) scores were available in half of the studies which provided quantitative data. It would have been possible also to have investigated the pooled effect on binary AUDIT outcome, though this was judged repetitious. All eight studies reported since 2005 provided unpublished data for inclusion in the meta analysis, with the authors of the earlier studies no longer having access to the raw data. These methods precluded certain forms of bias within studies, such as selective reporting of outcomes. All other outcomes were evaluated in a minority of available datasets. Outcomes 1 and 2 were converted into grams of ethanol [30].

Between-group mean differences in outcomes in the follow-up samples and their standard errors were calculated. Two trials had multiple follow-up intervals (at 1, 6 and 12, and 6 and 12 months respectively [31], [32]). Assessment effects were known to have been reported at 1 month and 12 months respectively and prior to analysis we decided that it was appropriately conservative to use the 6 month data as a summary measure in both studies to simplify the analyses. All data were meta-analysed in STATA version 10 with outcomes pooled in random effects models using the method of DerSimonian and Laird [33]. The I-squared statistic was used to evaluate the extent of heterogeneity [34].

## Results

Ten trials were identified for inclusion in this systematic review [31], [32], [35], [36], [37], [38], [39], [40], [41], [42], [43]. Two of these trials, including one study which reported outcomes separately by gender [39], [40], [41] did not provide findings for meta-analysis as outcome data were unreported and datasets were no longer accessible (see below). The characteristics of the included studies are presented in Table 1. One trial was not individually randomised, allocation being cluster randomised in weekly groups for each general practitioner, though it was not described as such because the terminology was not in common use at that time [35].

**Table 1:** Characteristics of studies included in meta-analyses.

	*Richmond et al. 1995*	*Kypri & McAnally 2005*	*Carey et al. 2006*	*Daeppen et al. 2007*	*Kypri et al. 2007*	*McCambridge & Day 2008*	*Walters et al. 2009*	*Cherpitel et al. 2010*
*Country*	Australia	New Zealand	USA	Switzerland	New Zealand	Britain	USA	Poland
*Setting*	General Practice	University primary healthcare clinic	University	Emergency Department (ED)	University primary healthcare clinic	University	University	Emergency Department (ED)
*Study design*	2 arm comparison nested within 4-arm trial	2 arm comparison nested within 3-arm trial	2 arm comparison nested within 6-arm trial	2 arm comparison nested within 3-arm trial	2 arm comparison nested within 4-arm trial	Dedicated 2-arm trial	2 arm comparison nested within 5-arm trial	2 arm comparison nested within 3-arm trial
*Population age, gender composition*	19–70 (mean 37.7); 43% women	17–24 years (mean 20.2); 49% women	18–25 years (mean 19.2); 67% women	18 years and over (mean 36.7); 22% women	17–29 years (mean 20.2); 52% women	18–24 years (mean 20.6); 67% women	18–38 years (mean 19.8); 66% women	18 years and over (39% <30 years); 16% women
*Eligibility/screening criteria*	>210/350 g ethanol per week for men/women	None	1+ episodes of heavy drinking (men: ≥5 drinks; women ≥4 drinks) in an average week or 4 episodes in the last month; class status not senior	Injury presentation to ED 11am–11pm, men under 65 years, 14 drinks per week and ≥5 drinks per session past 30 days, men over 65 years and women 7 drinks per week and ≥4 drinks per session past 30 days	Score of ≥8 on AUDIT	None	1+ episodes of heavy drinking (men: ≥5 drinks; women ≥4 drinks) in the preceding 2 weeks	Presentation to ED 4pm-midnight, RAPS4 positive screen or ≥11 drinks per week for men, >6 for women or >4 drinks for men per drinking day, >3 for women
*Exclusion of dependent drinkers*	Yes (physical dependence score>10 or MAST>20)	None	None	Yes, history of alcohol-related treatment in last 12 months	None	None	None	None
*Baseline sample size*	Control: 93Assessment: 93	Control: 72Assessment: 74	Control: 81Assessment: 89	Control: 335Assessment: 343	Control: 146Assessment: 147	Control: 204Assessment: 217	Control: 75Assessment: 72	Control: 147Assessment: 152
*Baseline drinking levels*	Drinks/weekControl: Mean 37.5 (SD 19.9)Assessment: Mean 34.7 (SD 18.2)	% binge drinkersControl: not assessedAssessment: 28% binge drinkers	Drinks/weekControl: Mean 19.3 (SD 11.2)Assessment: Mean 18.1 (SD 8.9)	a)Days drinking/wkb) Drinks per occasionControl: a) Mean 3.6 (SD 2.3)b) Mean 3.7 (SD 2.8)Assessment:a) Mean 3.5 (SD 2.4)b) Mean 3.8 (SD 2.4)	AUDIT scoreControl: Mean 15.1 (SD 5.5)Assessment: 14.9 (SD 5.0)	History of Trauma Scale positiveControl: 12%Assessment: 10%	Heavy drinking episodesControl: Mean 2.9 (SD 1.6)Assessment: Mean 3.3 (SD 1.9)	Drinks per drinking day*Control: Mean 5.5 (SE 0.4)Assessment: Mean 5.6 (SE 0.4)

*1/6 measures of consumption, dependence and prior treatment in both groups. Abbreviations: MAST = Michigan Alcohol Screening Test; AUDIT = Alcohol Use Disorders Identification Test.

Detailed information is presented in Table 2 on the assessment procedures being evaluated, blinding, and the consequent nature of the experimental contrasts employed. In some trials the experimental manipulations involved comparisons of longer versus shorter assessments [31], [32], whilst in others assessment was compared with minimal screening [35], [36], [37], or brief assessment with a screening instrument versus no screening at all [38]. The extent and nature of blinding and other potentially important aspects of study design were also variable across the studies. Table 3 comprises a summary of the primary study outcomes as they were reported. It is noteworthy that few of the statistically significant between-group differences attributed to answering questions are included in the present meta-analyses.

**Table 2:** Details of experimental contrasts.

	*Richmond et al. 1995*	*Kypri & McAnally 2005*	*Carey et al. 2006*	*Daeppen et al. 2007*	*Kypri et al. 2007*	*McCambridge & Day 2008*	*Walters et al. 2009*	*Cherpitel et al. 2010*
**Participants blind to…?**								
Study design	Not clear	Yes	Not clear	No	Yes	No	Yes	No
Group assignment	Not clear; judged likely	Yes	Not clear	No	Yes	Yes	Yes	No
Focus on drinking	Controls: YesAssessment group: No	Partially (other health behaviours assessed)	No	Partially (other health behaviours assessed)	No	Yes	No	No
Hypothesis	Yes	Yes	Not clear; judged likely	Not clear, judged likely	Yes	Yes	Yes	Not clear; judged likely
**Content of experimental conditions**								
Control	3-minute Health and Fitness Questionnaire: QF of drinking last 3 months, weight, smoking, exercise habits	Blood pressure measured, demographic details	Demographic details, height, weight, Daily Drinking Questionnaire, maximum number of drinks in last month and duration of episode, frequency of heavy drinking, RAPI	Screening only: 3 alcohol questions within a 10-item lifestyle questionnaire	Demographic details, AUDIT, number of drinks consumed in heaviest episode in last 4 weeks, +an 8-page leaflet on the effects of alcohol (online)	General health & sociodemographic questionnaire	Demographic questions only	RAPS-4 +3 questions on drinking (drinking days per week, drinks per average drinking day, maximum drinks in one occasion in past month
Assessment (As for controls +)	Drinking history; 7-day diary; MAST; physical dependence score	Age first drink, drank in last 12 months (Y/N), largest amount drunk in the last 4 weeks, AUDIT+non-alcohol measures	TLFB calendar for past 90 days: sequential assessment of alcohol use, drug use and sexual behaviour	AUDIT, 7-day TLFB+non-alcohol measures	4 weeks later: 14-day retrospective diary, APS, AREAS, perceived peer drinking norms	AUDIT	Alcohol consumption, related problems, protective behaviours, readiness to change, and perceived norms	Drinking in 6 hours before injury, feeling drunk at time of injury, attribution to alcohol, 30-day TLFB, SIP, readiness to change
Estimated times in minutes (control/assessment)	3/15	5/15	?/30	2/30	3/10	5/8	5/ 30 minutes (latter only repeated 3 m & 6 m)	3/10 (latter only repeated 3 m)
Medium of assessment administration	Not clear	Computer (Internet) self-completion	Face-to-face interview	Face-to-face interview	Computer (Internet) self-completion	Pen and paper self-completion	Computer (Internet) self-completion	Face-to-face interview
Non-alcohol content in assessment	None	Physical activity, fruit, vegetable consumption, smoking, mental health (from SF-36)	Drug use and sexual behaviour	Injury Severity Scale, presenting conditions, Quality of Life (SF-12)	None	None	None	Abbreviated Risk Taking/Impulsivity and Sensation Seeking Scales
Other features of assessment			Collateral interviews were conducted			Consent given for saliva sample		Breath testing for assessment group; List of AAgroups and treatment services given to all
Reimbursement/Payment	Not stated	All participants given a pen (value NZ$0.50) at invitation to follow-up	Paid US$20 and US$25 for 6 m and 12 m follow-up assessments.	None	Participants given sandwich voucher (NZ$4.95) when invited for follow-up	Paid £10 upon successful follow-up.	Psychology course credit or US$20 at baseline; US $20 at 3 and 6 mo. for assessment group only, US$40 at 12 mo. for all	Not stated

Abbreviations : MAST = Michigan Alcohol Screening Test; AUDIT = Alcohol Use Disorders Identification Test; APS = Alcohol Problems Scale; AREAS = Academic Role Expectations and Alcohol Scale; BAC = blood alcohol concentration; TLFB = Time Line Follow Back; RAPI = Rutgers Alcohol Problem Index; SF-12 = Short Form-12; SF-36 = Short Form-36; SIP = Short Index of Problems; RAPS-4 = Rapid Alcohol Problems Screen (4items).

**Table 3:** Study outcomes.

	*Richmond et al. 1995*	*Kypri & McAnally 2005*	*Carey et al 2006*	*Daeppen et al. 2007*	*Kypri et al. 2007*	*McCambridge & Day 2008*	*Walters et al. 2009*	*Cherpitel et al. 2010*
Numbers analysed	Control, Assessment6 m: 72, 66	Control, Assessment6 weeks: 61, 65	Control, Assessment1 m: 79, 886 m: 66, 6912 m: 59, 72	Control, Assessment12 m: 257, 277	Control, Assessment6 m: 124, 12212 m: 126, 126	Control, Assessment2–3 m: 144, 156	Control, Assessment12 m: 66, 63	Control, Assessment12 m: 91, 99
Outcome measures	Drinks last 7 days	• % Binge drinkers• Peak estimated BAC	• Drinks/week• Drinks/drinking day• Heavy drinking days• Peak estimated BAC previous month• RAPI	• Days drinking/week• Drinks/ drinking day• Number of binge drinking occasions• Drinks last 7 days• AUDIT• SF-12 Physical Comp. Score• SF-12 Mental Comp. Score	• Drinking days last 14 days• Drinks/drinking day• Drinks last 14 days• Heavy episodes last 14 days• APS• AREAS• AUDIT score (only at 12 m)	• AUDIT score• Drinks last 7 days• Drinking days last month• APS• AREAS• LDQ• % AUDIT>7• % 10+ drinks past 7 days• % Exceeded recommended weekly limit	• AUDIT score• % <8 on AUDIT• Drinks per week• Peak estimated BAC in previous month• Protective Behaviors Score including 4 subscale scores and 15 item scores	• RAPS-4• At-risk drinking (defined as for eligibility)• Drinking days per week• Drinks per drinking day• Maximum drinks per occasion• SIPS• Sought alcohol treatment
Duration of follow-up	6 m	6 weeks	1 m, 6 m, 12 m	12 m	6 m, 12 m	2–3 m	12 m	12 m
Summary of reported findings*	0/1 statistically significant differences	0/2 statistically significant differences	3/15 statistically significant differences:1 m• Drinks/wk• Drinks/drinking day• Peak BAC	0/6 statistically significant differences	4/13 statistically significant differences:12 m• Drinks last 14 days• Heavy episodes last 14 days• APS score• AUDIT	4/9 statistically significant differences:• AUDIT score• LDQ score• % AUDIT>7• % 10+ drinks past 7 days	7/25 statistically significant differences:• % <8 on AUDIT• Peak BAC• Protective Behaviors Score• Mixing beverages more weakly• Putting more ice in drink• Avoiding drinking games• Drinking slowly	0/7 statistically significant differences

*In all cases, statistical significance defined as p<0.05.

Abbreviations: APS = Alcohol Problems Scale; AREAS = Academic Role Expectations and Alcohol Scale; AUDIT = Alcohol Use Disorders Identification Test; BAC = blood alcohol concentration; LDQ = Leeds Dependency Questionnaire; RAPI = Rutgers Alcohol Problem Index; SF-12 = Short Form-12; SF-36 = Short Form-36; SIP = Short Index of Problems; RAPS-4 = Rapid Alcohol Problems Screen (4items).

**Figure 2:**
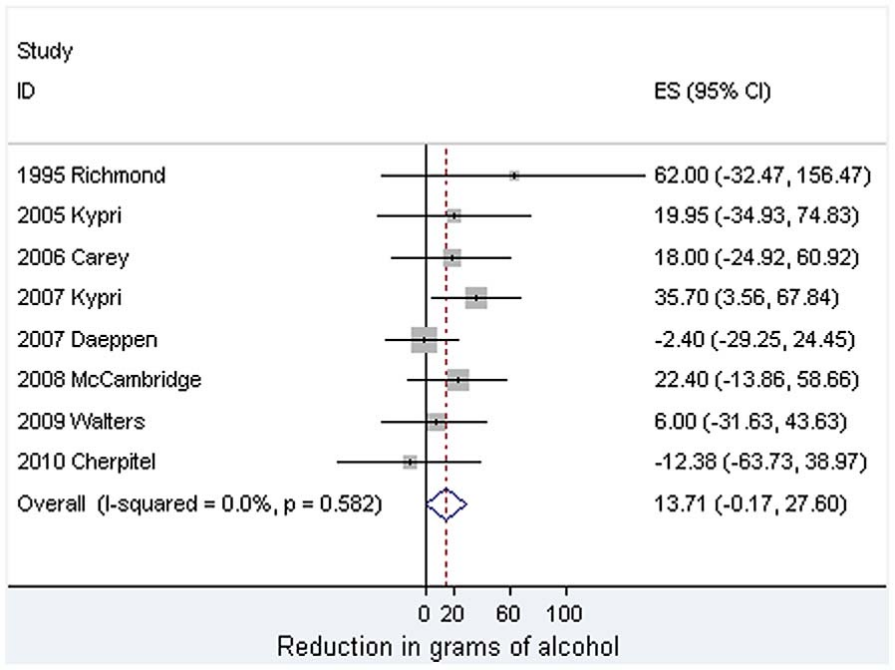
Meta-analysis of the effects of answering questions on total weekly drinking.

**Figure 3:**
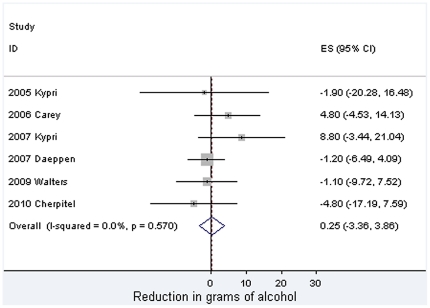
Meta-analysis of the effects of answering questions on quantity per drinking day.

**Figure 4:**
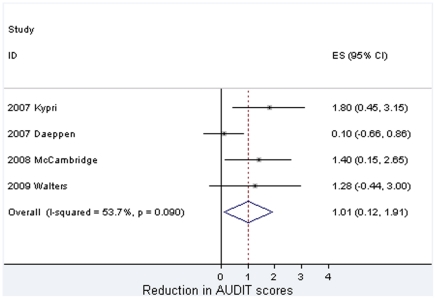
Meta-analysis of the effects of answering questions on total AUDIT scores.

Meta-analytic findings are presented in Figures 2, 3, 4. For past week alcohol consumption (Figure 2), the pooled effect marginally exceeds the 5% probability threshold (z = 1.94, p = 0.053) and is equivalent to approximately 1.5 UK units, and just over 1 standard drink in the USA [30]. No statistical heterogeneity is observed in relation to this effect. These studies are, however, clinically heterogeneous. Five studies took place in university student populations with pro-active recruitment of volunteers [31], [32], [36], [38], [42] and three among adults attending clinical services [35], [37], [43]. All but one [36] of the former reported effects of brief interventions on alcohol consumption, whereas none of the latter did. This lack of effectiveness in these latter studies makes them somewhat unusual in the literature on brief interventions in primary care where effectiveness has been established [22]. In the absence of differences between randomised brief intervention and unassessed control groups, it would be surprising if there were differences between assessed and unassessed control groups, the comparison of interest here. When this analysis is restricted to the five studies undertaken with university students, the pooled effect is greater and statistically significant (21.8 grams [4.4 to 39.2] difference, z = 2.46, p = 0.014). These findings contrast with the no difference found in quantity per drinking day (z = 0.14, p = 0.89) for which there was evidence of statistical heterogeneity (Figure 3).

The studies by Daeppen and colleagues [37] and Cherpitel and colleagues [43] both took place in Emergency Departments where evidence of brief intervention effectiveness is more uncertain than in general practice [44]. The Daeppen study also differs clearly from other studies in Figure 4, introducing heterogeneity and reducing the pooled estimate of effect from approximately 1.5 to 1 point (z = 2.21, p = 0.027). Twelve month AUDIT data from the study by Kypri and colleagues [32] were used because this outcome was not assessed at the 6-month follow-up interval. The three studies with the largest estimated effects on past week drinking all involved alcohol-only assessments [32], [35], [38].

The trial by Anderson and Scott reported findings separately for men and women [40], [41]. Both published reports of this trial contained the statement that “there were no significant differences between the control group who received no assessment and the group who received assessment” and provided no further outcome data. Statistical power to detect differences was limited. If it is assumed that retention was equivalent in the non-assessed group and the assessed group, estimated total sample sizes at follow-up were 46 women and 109 men. Among men this provides approximately only 18% power to detect a small effect of 0.2 standard deviations. The trial by Gentilello and colleagues [39] did not refer to relevant outcomes in the published report. E-mail contact with the lead author ascertained that analyses had been undertaken and no differences in outcome detected between assessed and non-assessed groups (L. Gentillelo, personal communication). Again assuming that attrition was not different between the randomised groups, this sample of 307 provides approximately 41% power to detect a difference of 0.2 standard deviations.

## Discussion

Ten trials of the effects of answering questions in research assessment procedures within brief alcohol intervention studies were identified. Outcome data were pooled on three specific measures of drinking behaviour drawn from eight trials with data available. This revealed somewhat equivocal evidence of small effects on two of the three outcomes across the studies as a whole. Evidence of assessment reactivity appears stronger if one restricts attention to the student literature. The possible effect on past weekly total consumption was not detected to a statistically significant level in any of the eight primary studies. This pattern of findings is behaviourally plausible where reduced overall alcohol consumption is caused by less frequent drinking with a consequent reduction in risk as assessed with the AUDIT, with quantity per drinking occasion remaining unaltered.

Five of the eight studies included in the meta-analysis were undertaken in healthcare settings and five of the studies involved university students, with two studies taking place in student health services [32], [36]. The generalisability of these findings thus warrants careful scrutiny. This point is also reinforced by the detection of the two unpublished studies in general medical settings with no statistically significant effects, which incidentally gives reason for confidence in the completeness of our identification methods. Whilst the study by Anderson and Scott [40], [41] had very limited capacity to detect effects, this was much less true of the study by Gentilello and colleagues [39].

The methodological quality of included studies has not been formally assessed, making caution further necessary, as biases in trials will produce biased pooled effect estimates in meta-analyses. For example, though attrition is generally low, it is higher in some studies than in others and even small differences between groups across studies may introduce bias. We chose a single follow-up interval in the studies by Carey et al. [31] and Kypri et al. [32] and eschewed evaluation of binary AUDIT outcome which was statistically significant in the study by Walters and colleagues [42] in favour of continuous AUDIT score which was not. Close inspection of the data in the tables and figures suggests that both these decisions lead towards more conservative estimates of the effects of answering questions. We also deliberately ignored statistically significant effects within the primary studies on outcomes which have not been employed consistently across the studies.

Findings of a small effect on drinking behaviour are coherent with data on various other outcomes in the relatively few individual trials that exist in the wider health sciences [13], [14], [15], [16], [17], [18], [20], [21]. These too have generally identified small effects, though they include one study which found no effects [20], and also one study which identified a large effect [19]. This pattern is found also in the wider non-health social science literature. For example prior questionnaire completion exerts a measurable small effect on voting behaviour [45], [46], as well as in laboratory-based social psychology experiments [47].

The outcome data in the present study were all self-reported, necessarily so given the target behaviour, and other investigations of this phenomenon also rely on such data [13], [14], [15], [18]. Importantly, however, studies do exist which identify effects of similar magnitude upon objectively assessed behaviours [16], [17], [21]. For example, the large effect obtained in a dental study was on plaque coverage ascertained using photography [19]. Furthermore, Godin and colleagues [17] observed both registrations at blood drives and blood donations, neither requiring self-report data, and effects have also been detected on attendances for screening in other studies [16], [21]. Intriguingly, a large effect on the amount of money deposited in an honesty box was also unobtrusively obtained in an experiment stimulating a sense of being observed [48].

Given the under-development of this area of study, there are many potential sources of bias which remain to be investigated to permit clear and confident causal inferences. Notwithstanding these cautionary remarks, what are the implications of the findings from the present study? The magnitude of an apparent assessment effect quantified here is small, though that should not be interpreted to mean that it is unimportant. The difference in past week consumption represents approximately 35% of the known effect of brief alcohol intervention in primary care [22]. As assessment is an integral component of a brief intervention, contamination has occurred, attenuating estimates of intervention effects. Brief interventions may thus be much more effective than has been previously understood. The present study needs to be replicated when the literature has further developed and the equivocal nature of the overall findings and the apparent discrepancies between student and non-student populations resolved. There is also a need to study whether answering questions on other behaviours generates similar reactivity in trials. The present findings suggest this form of contamination may be more common than has previously been appreciated.

The small effects potentially attributable here to answering questions have been detected as unwanted artefacts of the research process. Almost all these questions are concerned with measurement of behaviour and its consequences. These questions have thus not been designed to elicit thinking about change, and thus to promote actual behaviour change. This is true also of the wider literature with the exception of the studies by Sandberg and Conner [21] and Godin and colleagues [17], in which questions specifically about anticipated regret and implementation intentions were asked. It is likely that selecting questions for their behaviour change potential may produce greater effects than have been seen here.

Answering questions appears to exert a subtle influence on subsequent self-reported drinking behaviour among students. Other aspects of the research process such as randomisation [49], [50], [51] and consent [52], [53] also have psychological impacts. Their implications for subsequent behaviour remain to be evaluated [54], and may cumulatively generate greater bias. This impairs our ability to rule out reactivity to the research conditions themselves as a possible explanation for observed between-group differences in trials, thereby impeding secure inferences on the true effects of behavioural interventions [55]. These uncertainties are ironically produced by the unintended and largely overlooked consequences of undertaking research itself. Whilst behavioural science has had some awareness of these issues for some considerable time, rapid advances in understanding are now well overdue [56].

## References

[1] Lopez AD, Mathers CD, Ezzati M, Jamison DT, Murray CJL (2006). Global and regional burden of disease and risk factors, 2001: Systematic analysis of population health data.. Lancet.

[2] Rosnow RL, Rosenthal R (1997). People Studying People: Artifacts and Ethics in Behavioral Research.

[3] Solomon RL (1949). An extension of control group design.. Psychol Bull.

[4] Mayo E (1933). The human problems of an industrial civilization.

[5] Roethlisberger FJ, Dickson WJ (1939). Management and the Worker.

[6] Gillespie R (1991). Manufacturing knowledge: a history of the Hawthorne experiments.

[7] Parsons HM (1974). What happened at Hawthorne?. Science.

[8] Kinmonth AL, Wareham NJ, Hardeman W, Sutton S, Prevost AT (2008). Efficacy of a theory-based behavioural intervention to increase physical activity in an at-risk group in primary care (ProActive UK): a randomised trial.. Lancet.

[9] Clemes SA, Parker RA (2009). Increasing our understanding of reactivity to pedometers in adults.. Med Sci Sports Exerc.

[10] Campbell JP, Maxey VA, Watson WA (1995). Hawthorne effect: implications for prehospital research.. Ann Emerg Med.

[11] Eckmanns T, Bessert J, Behnke M, Gastmeier P, Ruden H (2006). Compliance with antiseptic hand rub use in intensive care units: the Hawthorne effect.. Infect Control Hosp Epidemiol.

[12] Carabin H, Gyorkos TW, Soto JC, Joseph L, Payment P (1999). Effectiveness of a training program in reducing infections in toddlers attending day care centers.. Epidemiology.

[13] Bouchet C, Guillemin F, Briancon S (1996). Nonspecific effects in longitudinal studies: impact on quality of life measures.. Journal of Clinical Epidemiology.

[14] DeAmici D, Klersy C, Ramajoli F, Brustia L, Politi P (2000). Impact of the Hawthorne effect in a longitudinal clinical study: the case of anaesthesia.. Controlled Clinical Trials.

[15] McCarney R, Warner J, Iliffe S, van Haselen R, Griffin M (2007). The Hawthorne Effect: a randomised, controlled trial.. BMC Med Res Methodol.

[16] O'Sullivan I, Orbell S, Rakow T, Parker R (2004). Prospective research in health service settings: health psychology, science and the ‘Hawthorne’ effect.. J Health Psychol.

[17] Godin G, Sheeran P, Conner M, Germain M (2008). Asking questions changes behavior: mere measurement effects on frequency of blood donation.. Health Psychol.

[18] Clifford PR, Maisto SA, Davis CM (2007). Alcohol treatment research assessment exposure subject reactivity effects part I. Alcohol use and related consequences.. Journal of Studies on Alcohol and Drugs.

[19] Feil PH, Grauer JS, Gadbury-Amyot CC, Kula K, McCunniff DDS (2002). Intentional use of the Hawthorne Effect to improve oral hygiene compliance in orthodontic patients.. Journal of Dental Education.

[20] del Junco DJ, Vernon SW, Coan SP, Tiro JA, Bastian LA (2008). Promoting regular mammography screening I. A systematic assessment of validity in a randomized trial.. J Natl Cancer Inst.

[21] Sandberg T, Conner M (2009). A mere measurement effect for anticipated regret: impacts on cervical screening attendance.. Br J Soc Psychol.

[22] Kaner EF, Beyer F, Dickonson HO, Pienaar E, Campbell F (2007). Effectiveness of brief alcohol interventions in primary care populations.. Cochrane Database of Systematic Reviews:.

[23] Bernstein JA, Bernstein E, Heeren TC (2010). Mechanisms of change in control group drinking in clinical trials of brief alcohol intervention: implications for bias toward the null.. Drug Alcohol Rev.

[24] McCambridge J (2009). [Commentary] Research assessments: instruments of bias and brief interventions of the future?. Addiction.

[25] Moher D, Liberati A, Tetzlaff J, Altman DG (2009). Preferred reporting items for systematic reviews and meta-analyses: the PRISMA statement.. BMJ.

[26] Babor T, Lauerman R, Kranzler H, McRee B, Korner P (1992). Farmington, USA. Project on Identification and Management of Alcohol-Related Problems Report on Phase II: a Randomized Clinical Trial of Brief Interventions in Primary Care.

[27] Serrano C, Martinez D, Mendoza M, Sanchez S, Velasquez J (1992). Mexico City, Mexico. Project on Identification and Management of Alcohol-Related Problems Report on Phase II: a Randomized Clinical Trial of Brief Interventions in Primary Care.

[28] Kuntsche E, Robert B (2009). Short message service (SMS) technology in alcohol research–a feasibility study.. Alcohol Alcohol.

[29] Babor TF, Higgin-Biddle JC, Saunders JB, Monteiro MG (2001). The Alcohol Use Identification Test: Guidelines for Use in Primary Care. 2nd Edition.

[30] Miller WR, Heather N, Hall W (1991). Calculating standard drink units: International comparisons.. British Journal of Addiction.

[31] Carey KB, Carey MP, Maisto SA, Henson JM (2006). Brief motivational interventions for heavy college drinkers: A randomized controlled trial.. Journal of Consulting and Clinical Psychology.

[32] Kypri K, Langley JD, Saunders JB, Cashell-Smith ML (2007). Assessment may conceal therapeutic benefit: findings from a randomized controlled trial for hazardous drinking.. Addiction.

[33] DerSimonian R, Laird N (1986). Meta - analysis in clinical trials.. Controlled Clinical Trials.

[34] Higgins JPT, Thompson SG (2002). Quantifying heterogeneity in meta - analysis.. Statistics in Medicine.

[35] Richmond RL, Heather N, Wodak A, Kehoe L, Webster I (1995). Controlled evaluation of a general practice-based brief intervention for excessive drinking.. Addiction.

[36] Kypri K, McAnally HM (2005). Randomized controlled trial of a web-based primary care intervention for multiple health risk behaviors.. Prev Med.

[37] Daeppen JB, Gaume J, Bady P, Yersin B, Calmes JM (2007). Brief alcohol intervention and alcohol assessment do not influence alcohol use in injured patients treated in the emergency department: a randomized controlled clinical trial.. Addiction.

[38] McCambridge J, Day M (2008). Randomized controlled trial of the effects of completing the Alcohol Use Disorders Identification Test questionnaire on self-reported hazardous drinking.. Addiction.

[39] Gentilello L, Rivara E, Donovan D, Jurkovich G, Daranciang E (1999). Alcohol interventions in a trauma center as a means of reducing the risk of injury recurrence.. Annals of Surgery.

[40] Scott E, Anderson P (1990). Randomised controlled trial of general practitioner intervention in women with excessive alcohol consumption.. Drug and Alcohol Review.

[41] Anderson P, Scott E (1990). The effect of general practitioners' advice to heavy drinking men.. British Journal of Addiction.

[42] Walters ST, Vader AM, Harris TR, Jouriles EN (2009). Reactivity to alcohol assessment measures: an experimental test.. Addiction.

[43] Cherpitel CJ, Korcha RA, Moskalewicz J, Swiatkiewicz G, Ye Y (2010). Screening, brief intervention, and referral to treatment (SBIRT): 12-month outcomes of a randomized controlled clinical trial in a Polish emergency department.. Alcohol Clin Exp Res.

[44] Havard A, Shakeshaft A, Sanson-Fisher R (2008). Systematic review and meta-analyses of strategies targeting alcohol problems in emergency departments: interventions reduce alcohol-related injuries.. Addiction.

[45] Voogt RJJ, VanKempen H (2002). Nonresponse bias and stimulus effects in the Dutch National Election Study.. Quality & Quantity.

[46] Greenwald AG, Carnot CG, Beach R, Young B (1987). Increasing voting behaviour by asking young people if they expect to vote.. Journal of Applied Psychology.

[47] Fitzsimons GJ, Moore SG (2008). Should we ask our children about sex, drugs and rock & roll? Potentially harmful effects of asking questions about risky behaviors.. Journal of Consumer Psychology.

[48] Bateson M, Nettle D, Roberts G (2006). Cues of being watched enhance cooperation in a real-world setting.. Biol Lett.

[49] Cook TD, Campbell DT (1979). Quasi-Experimentation: Design and Analysis. Issues for Field Settings.

[50] Brewin CR, Bradley C (1989). Patient preferences and randomized clinical trials.. British Medical Journal.

[51] King M, Nazareth I, Lampe F, Bower P, Chandler M (2005). Impact of participant and physician intervention preferences on randomized trials: a systematic review.. JAMA.

[52] Zelen M (1990). Randomized consent designs for clinical trials: an update.. Statistics in Medicine.

[53] Adamson J, Cockayne S, Puffer S, Torgerson DJ (2006). Review of randomised trials using the post-randomised consent (Zelen's) design.. Contemporary Clinical Trials.

[54] Kypri K, McCambridge J, Wilson A, Attia J, Sheeran P (2011). Effects of Study Design and Allocation on participant behaviour - ESDA: study protocol for a randomized controlled trial.. Trials.

[55] McCambridge J, Butor-Bhavsar K, Witton J, Elbourne D (in press). Can research assessments themselves cause bias in behaviour change trials? A systematic review of evidence from Solomon 4-group studies.. PLoS ONE.

[56] McCambridge J, Kypri K, Elbourne DR (2009). A surgical safety checklist.. N Engl J Med.

